# Presentation, Management, and In-Hospital Outcomes of Patients with Acute Heart Failure in South India by Sex: A Secondary Analysis of a Prospective, Interrupted Time Series Study

**DOI:** 10.5334/gh.1043

**Published:** 2021-09-27

**Authors:** Anubha Agarwal, Padinhare P. Mohanan, Dimple Kondal, Aashima Chopra, Abigail S. Baldridge, Divin Davies, Raji Devarajan, Govindan Unni, Jabir Abdullakutty, Syam Natesan, Johny Joseph, Pathiyil B. Jayagopal, Stigi Joseph, Rajesh Gopinath, Dorairaj Prabhakaran, Mark D. Huffman

**Affiliations:** 1 Department of Medicine, Northwestern University Feinberg School of Medicine, Chicago, IL, US; 2 WestFort Hi-Tech Hospital, Thrissur, Kerala, IN; 3 Centre for Chronic Disease Control, New Delhi, Delhi, IN; 4 Department of Preventive Medicine, Northwestern University Feinberg School of Medicine, Chicago, IL, US; 5 Jubilee Mission Medical College and Hospital, Thrissur, Kerala, IN; 6 Lisie Hospital, Kochi, Kerala, IN; 7 Kollam District Hospital, Kollam, Kerala, IN; 8 Caritas Hospital, Kottayam, Kerala, IN; 9 Lakshmi Hospital, Palakkad, Kerala, IN; 10 Little Flower Hospital and Research Centre, Angamaly, Kerala, IN; 11 Amala Institute of Medical Sciences, Thrissur, Kerala, IN; 12 The Public Health Foundation of India, Gurugram, Haryana, IN; 13 The George Institute for Global Health, University of New South Wales, Sydney, New South Wales, AU

**Keywords:** Heart failure, Heart failure with reduced ejection fraction, Guideline-directed medical therapy, Sex-specific differences, India

## Abstract

**Background:**

Sex differences in presentation, management, and outcomes of heart failure (HF) have been observed, but it is uncertain whether these differences exist in South India.

**Objective:**

We describe sex differences in presentation, management, and in-hospital outcomes in patients hospitalized with HF in South India and explore sex-based differences in the effect of the quality improvement intervention in a secondary analysis of a prospective, interrupted time series study.

**Methods:**

The Heart Failure Quality Improvement in Kerala (HF QUIK) study evaluated the effect of a quality improvement toolkit on process of care measures and clinical outcomes in patients hospitalized with HF in eight hospitals in Kerala using an interrupted time series design from February 2018 to August 2018. The primary outcome was guideline-directed medical therapy (GDMT) at hospital discharge for patients with HF with reduced ejection fraction (HFrEF). We performed sex-stratified analyses using mixed effect logistic regression models.

**Results:**

Among 1,400 patients, 536 (38.3%) were female. Female patients were older (69.6 vs. 65 years, p < 0.001), were less likely to have an ischemic etiology of HF (control period: 78.2% vs. 87.5%; intervention period: 83.6% vs. 91.5%; p < 0.05 for both) and were less likely to undergo coronary angiography or percutaneous coronary intervention. The quality improvement intervention had similar effects on the odds of GDMT at discharge in females with HFrEF (adjusted OR 1.79, 95% CI 0.92, 3.47) and males with HFrEF (adjusted OR 1.68, 95% CI 1.07, 2.64, p_interaction_ = 0.69).

**Conclusions:**

We observed sex-specific differences in presentation and procedural management of patients with HF but no differences in the effect of the quality improvement intervention on discharge GDMT rates. Both male and female patients with HFrEF remained undertreated in the study intervention period, demonstrating the need for implementation strategies to close the HFrEF treatment gap in South India.

## Introduction

Heart failure (HF) is a leading global public health problem. There are more than 64 million (95% uncertainty interval [UI]: 57 to 72) people estimated to have HF globally with higher prevalence rates among females (35 million, 95% UI: 31 to 39) compared to males (30 million, 95% UI: 26 to 33) [1]. The burden of HF is increasingly shifting from high-income to low- and middle-income countries due to population growth, aging, and increasing prevalence of major HF risk factors [1,2,3,4,5,6,7]. Data from global randomized HF trials suggests persistent sex-based differences in experience and treatment of patients with HF with reduced ejection fraction (HFrEF) [8]. Less is known about sex-based differences in HF in Asia where nearly half of the increase in number of global HF cases from 1990 to 2017 occurred in China and India [1]. Observational registry data in India demonstrates no significant sex-based differences between males and females with acute coronary syndromes but highlight sex-based differences in cardiovascular disease in India are understudied [9,10].

Despite increasing international attention to the importance of sex-based differences in cardiovascular disease, there has been no significant proportional growth in sex-specific publications in the past decade based on a bibliometric analysis of all cardiovascular publications from 2006 to 2015 [11]. To address this gap in India, we performed a secondary post-hoc analysis of the Heart Failure Quality Improvement in Kerala (HF QUIK) study to describe the presentation, management, and in-hospital outcomes of males and females hospitalized with HF in South India and explore sex-based differences in the effect of the quality improvement intervention.

## Methods

### Study design, context, and participants

The HF QUIK study was a prospective, interrupted time series study that evaluated the effect of a quality improvement intervention on process of care measures and clinical outcomes in patients hospitalized with HF in eight hospitals in South India from February 2018 to August 2018 [12]. Detailed methods and overall results have been published. In brief, we recruited eight hospitals in Kerala from a sampling frame of 63 hospitals that had participated in the Acute Coronary Syndrome Quality Improvement in Kerala (ACS QUIK) trial [13]. The eight participating hospitals represented diverse implementation settings including government, non-profit/charity, and private hospitals. Furthermore, these participating hospitals were spread geographically across the state (northern, central, southern regions) including rural and urban settings. Patients admitted with a primary diagnosis of HF were enrolled consecutively at each hospital site. Patients were eligible for inclusion if they were: 1) adults aged 18 years or older, and 2) met at least two criteria for diagnosis of HF as defined by the European Society of Cardiology (e.g., clinical symptoms and signs of HF, natriuretic peptide elevation, or echocardiographic evidence of left ventricular systolic or diastolic dysfunction) [14]. These inclusion criteria were analogous to those used in HF registries in the region [15,16,17,18].

Study coordinators at each hospital site entered data into an electronic data capture tool (REDCap, Vanderbilt University, Nashville, TN, USA) [19]. The study protocol received ethics board approval from Duke University (Durham, NC, USA), Centre for Chronic Disease Control (New Delhi, Delhi, India), Cardiological Society of India-Kerala Chapter (Kochi, Kerala, India), and Indian Health Ministry Screening Committee (New Delhi, Delhi, India) in November 2017. Hospital sites were granted a waiver of informed consent under the Common Rule because data were used at local hospitals for the purpose of quality improvement.

### Quality improvement intervention and outcomes

Formative research including a systematic review of quality improvement interventions for patients admitted with HF, gaps in quality of care identified by HF registries in the region, and key informant in-depth interviews contributed to the design of the HF QUIK quality improvement intervention [15,16,17,20,21]. The HF QUIK quality improvement intervention consisted of in-hospital and discharge checklists to prompt healthcare providers to order guideline-recommended in-hospital diagnostics (e.g., electrocardiogram, natriuretic peptide, transthoracic echocardiogram), prescribe guideline-directed medical therapy (GDMT), conduct patient education for HF-specific health behaviors, and pursue post-discharge recommendations (e.g., referral for implantable cardioverter defibrillator or cardiac resynchronization therapy in eligible patients, referral for outpatient cardiac rehabilitation, schedule follow-up in outpatient clinic). Healthcare providers were given patient education materials written in the local language of Malayalam on healthy behaviors, including diet, activity, and alcohol and smoking cessation. Hospital sites received personalized audit-and-feedback reports emphasizing site-specific performance measures based on established HF quality metrics [22]. Each hospital site received on-site training of the quality improvement intervention with the hospital investigator, site study coordinator, cardiac care unit and general cardiology ward nurses. The pre-intervention control period consisted of usual care according to local hospital practice.

The primary outcome was the prescription of GDMT at discharge (e.g., angiotensin-converting enzyme inhibitor [ACE-I] or angiotensin receptor blocker [ARB], beta-blocker, and aldosterone antagonist) for patients with HFrEF (defined as ejection fraction less than 40%) measured separately and as a combined outcome. Secondary outcomes included in-hospital process of care measures (e.g., electrocardiogram, transthoracic echocardiogram), discharge process of care measures (e.g., tobacco and alcohol cessation counseling, diet counseling, weight monitoring instructions, referral for outpatient cardiac rehabilitation, referral for implantable cardioverter defibrillator or cardiac resynchronization therapy, outpatient follow-up appointment scheduled at discharge) and clinical outcomes (e.g., inpatient mortality).

### Statistical analysis

For this report, we stratified analyses by sex. We used date of admission to allocate participants to the control period or the intervention period. Baseline characteristics are summarized for control and intervention periods by sex. Continuous variables are reported as means with standard deviations or medians with interquartile range if data were skewed, and categorical variables as counts with percentages. Between-group comparisons were made using Student’s *t* test and Chi-square test for continuous and categorical variables, respectively. We evaluated the odds of adherence to process of care measures pre- and post-intervention overall and between sex groups through logistic regression models. The unadjusted model includes the group variable (control and intervention period). The adjusted mixed effect logistic regression model was adjusted for age, and we included a random effect model to account for within-hospital clustering. We performed a complete case analysis due to low rate of missing data (0.1%). Two-sided p value < 0.05 was used to define statistical significance. We used Stata version 14 (Stata Corp, College Station, TX, USA) for statistical analyses.

## Results

We enrolled 1,469 patients from 8 hospitals in Kerala, India. Patients were excluded if there were duplicate data entries for the same patient (n = 13), had missing data (n = 2), or were admitted to the hospital after the final date of study enrollment (n = 54). The complete case analysis was performed on 1,400 patients with 758 patients (n = 441 male, n = 317 female) in the control period and 642 patients (n = 423 male, n = 219 female) in the intervention period (Supplementary Appendix Figure 1).

Sex-stratified baseline characteristics of study patients in control and intervention periods are shown in Table 1. Of the 1,400 patients, 536 (38.3%) were female. Female patients were older with mean (SD) age of 68.7 (12.5) years in control period and 69.6 (12.2) years in intervention period. Female patients were less likely to have an ischemic etiology of HF compared to male patients (control period: 78.2% vs. 87.5%; intervention period: 83.6% vs. 91.5%; p < 0.05 for both). Female patients reported lower tobacco and alcohol use compared to male patients. Male patients had higher rates of chronic kidney disease (control period: 20.9% vs 11.7%; intervention period: 18.2% vs. 9.1%; p < 0.05 for both) and higher median creatinine on admission compared to female patients. Female patients had higher left ventricular ejection fraction compared to male patients (control period: 37.1% vs. 34.5%; intervention period: 36.6% vs. 33.9%; p < 0.001 for both). Table 2 shows in-hospital diagnostics, procedures, and treatment stratified by sex in control and intervention periods. Female patients were less likely to undergo coronary angiography (control period: 15.1% vs. 26.1%; intervention period: 11.4% vs. 25.5%; p < 0.001 for both) and percutaneous coronary intervention. There were no major differences between in-hospital treatment of male and female patients.

**Table 1:** Baseline characteristics of HF QUIK participants in control and intervention periods by sex.

Participant characteristics	Control		P-value	Intervention		P-value
	
	Male N = 441 n (%)	Female N = 317 n (%)		Male N = 423 n (%)	Female N = 219 n (%)	

Age, mean (SD), y	65.3 (12.1)	68.7 (12.5)	<0.001	65.0 (11.5)	69.6 (12.2)	<0.001
Transferred from another facility	168 (38.1)	114 (36.0)	0.800	150 (35.5)	75 (34.2)	0.560
Ischemic etiology of HF	386 (87.5)	248 (78.2)	0.002	387 (91.5)	183 (83.6)	0.011
**Self-reported medical history prior to HF admission**						

Tobacco use	260 (59.0)	7 (2.2)	<0.001	231 (54.6)	2 (0.9)	<0.001
Alcohol use	183 (41.5)	4 (1.3)	<0.001	153 (36.2)	1 (0.5)	<0.001
Coronary heart disease	269 (61.0)	140 (44.2)	<0.001	257 (60.8)	120 (54.8)	0.150
Percutaneous coronary intervention	50 (11.3)	17 (5.4)	0.004	51 (12.1)	11 (5.0)	0.004
Diabetes mellitus	234 (53.1)	194 (61.2)	0.026	206 (48.7)	133 (60.7)	0.004
Hypertension	241 (54.6)	206 (65.0)	0.004	239 (56.5)	132 (60.3)	0.360
Hyperlipidemia	80 (18.1)	81 (25.6)	0.014	92 (21.7)	42 (19.2)	0.450
Valvular heart disease	28 (6.3)	24 (7.6)	0.510	17 (4.0)	19 (8.7)	0.015
Rheumatic heart disease	11 (2.5)	13 (4.1)	0.210	6 (1.4)	15 (6.8)	<0.001
Chronic kidney disease	92 (20.9)	37 (11.7)	<0.001	77 (18.2)	20 (9.1)	0.002
Stroke	31 (7.0)	23 (7.3)	0.910	33 (7.8)	9 (4.1)	0.073
Implantable cardioverter defibrillator	5 (1.1)	0 (0.0)	0.057	4 (0.9)	0 (0.0)	0.150
Cardiac resynchronization therapy	3 (0.7)	2 (0.6)	0.930	3 (0.7)	1 (0.5)	0.700
**Medications prior to HF admission**						

Loop diuretic	181 (41.0)	116 (36.6)	0.220	161 (38.1)	97 (44.3)	0.130
Thiazide diuretic	7 (1.6)	1 (0.3)	0.091	3 (0.7)	5 (2.3)	0.088
ACE-I or ARB	99 (22.5)	80(25.2)	0.373	92 (21.8)	64 (29.2)	0.036
Beta-blocker	142 (32.2)	91 (28.7)	0.300	163 (38.5)	84 (38.4)	0.960
Aldosterone antagonist	88 (20.0)	47 (14.8)	0.069	58 (13.7)	29 (13.2)	0.870
ARNi	7 (1.6)	3 (0.9)	0.450	3 (0.7)	0 (0.0)	0.210
Digoxin	49 (11.1)	29 (9.1)	0.380	27 (6.4)	25 (11.4)	0.027
Ivabradine	17 (3.9)	17 (5.4)	0.320	15 (3.5)	5 (2.3)	0.380
Aspirin	210 (47.6)	124 (39.1)	0.020	208 (49.2)	110 (50.2)	0.800
Statin	211 (47.8)	136 (42.9)	0.180	214 (50.6)	116 (53.0)	0.570
**Physical exam, laboratory and imaging**						

Weight, mean (SD), kg	65.8 (11.7)	60.3 (10.2)	<0.001	66.4 (10.1)	60.2 (10.4)	<0.001
Systolic blood pressure, mean (SD), mmHg	137.6 (29.9)	140.2 (30.5)	0.240	141.2 (31.1)	141.7 (29.5)	0.850
Diastolic blood pressure, mean (SD), mmHg	82.9 (16.4)	81.7 (14.1)	0.320	83.7 (16.3)	83.2 (13.8)	0.740
Heart rate, mean (SD), bpm	91.4 (23.0)	96.4 (23.4)	0.003	94.3 (23.2)	93.7 (24.9)	0.750
Sodium, mean (SD), mEq/L	135.0 (5.9)	134.1 (6.7)	0.067	134.9 (5.2)	134.4 (4.9)	0.230
Creatinine, median (IQR), mg/dL	1.3 (1.0, 1.8)	1.1 (0.9, 1.5)	<0.001	1.3 (1.0, 1.8)	1.1 (0.9, 1.6)	<0.001
Ejection fraction, mean (SD), %	34.5 (9.3)	37.1 (10.5)	<0.001	33.9 (9.1)	36.6 (10.2)	<0.001

ACE-I: angiotensin converting enzyme inhibitor, ARB: angiotensin receptor blocker, ARNi: angiotensin receptor neprilysin inhibitor, IQR: interquartile range.

**Table 2:** In-hospital tests, procedures, and treatment of HF QUIK participants in control and intervention periods by sex.

Diagnostic tests and treatment	Control		P-value	Intervention		P-value
	
	Male N = 441 n (%)	Female N = 317 n (%)		Male N = 423 n (%)	Female N = 219 n (%)	

**In-hospital tests and procedures**						

ECG, No. (%)	436 (98.9)	315 (99.4)	0.48	423 (100.0)	218 (99.5)	0.16
Cardioversion, No. (%)	16 (3.6)	7 (2.2)	0.26	11 (2.6)	3 (1.4)	0.31
Stress testing, No. (%)	0 (0.0)	1 (0.3)	0.24	0	0	
Coronary angiography, No. (%)	115 (26.1)	48 (15.1)	<0.001	108 (25.5)	25 (11.4)	<0.001
Percutaneous coronary intervention, No. (%)	34 (7.7)	11 (3.5)	0.015	39 (9.2)	5 (2.3)	<0.001
Coronary artery bypass graft, No. (%)	3 (0.7)	3 (0.9)	0.68	7 (1.7)	2 (0.9)	0.45
Implantable cardioverter defibrillator, No. (%)	3 (0.7)	1 (0.3)	0.49	3 (0.7)	0 (0.0)	0.21
Cardiac resynchronization therapy, No. (%)	1 (0.2)	0 (0.0)	0.40	2 (0.5)	1 (0.5)	0.98
Intra-aortic balloon pump, No. (%)	0	0		1 (0.2)	0 (0.0)	0.47
Dialysis or ultrafiltration, No. (%)	4 (0.9)	4 (1.3)	0.64	8 (1.9)	1 (0.5)	0.14
Non-invasive positive pressure ventilation, No. (%)	110 (24.9)	108 (34.1)	0.006	112 (26.5)	112 (26.5)	0.71
Mechanical ventilation, No. (%)	39 (8.8)	29 (9.1)	0.88	39 (9.2)	18 (8.2)	0.67
**In-hospital treatment**						

Loop diuretic, No. (%)	411 (93.2)	302 (95.3)	0.23	415 (98.1)	216 (98.6)	0.63
Thiazide diuretic, No. (%)	8 (1.8)	8 (2.5)	0.50	10 (2.4)	6 (2.7)	0.77
ACE-I or ARB, No. (%)	170 (38.6)	140 (44.2)	0.121	173 (40.9)	110 (50.2)	0.024
Beta-blocker, No. (%)	317 (71.9)	225 (71.0)	0.79	317 (74.9)	161 (73.5)	0.69
Aldosterone antagonist, No. (%)	259 (58.7)	177 (55.8)	0.43	278 (65.7)	153 (69.9)	0.29
ARNi, No. (%)	11 (2.5)	6 (1.9)	0.58	8 (1.9)	0 (0.0)	0.041
Digoxin No. (%)	90 (20.4)	63 (19.9)	0.86	62 (14.7)	45 (20.5)	0.058
Ivabradine, No. (%)	42 (9.5)	36 (11.4)	0.41	49 (11.6)	21 (9.6)	0.44
Aspirin, No. (%)	376 (85.3)	256 (80.8)	0.10	377 (89.1)	179 (81.7)	0.009
Statin, No. (%)	374 (84.8)	264 (83.3)	0.57	379 (89.6)	180 (82.2)	0.008
Hydralazine-nitrate, No. (%)	28 (6.3)	17 (5.4)	0.57	16 (3.8)	7 (3.2)	0.70
Nitroglycerin, No. (%)	129 (29.3)	90 (28.4)	0.80	137 (32.4)	67 (30.6)	0.64
Inotrope, No. (%)	81 (18.4)	48 (15.1)	0.24	92 (21.7)	34 (15.5)	0.06

ECG: electrocardiogram, ACE-I: angiotensin converting enzyme inhibitor, ARB: angiotensin receptor blocker, ARNi: angiotensin receptor neprilysin inhibitor.

**Figure 1:**
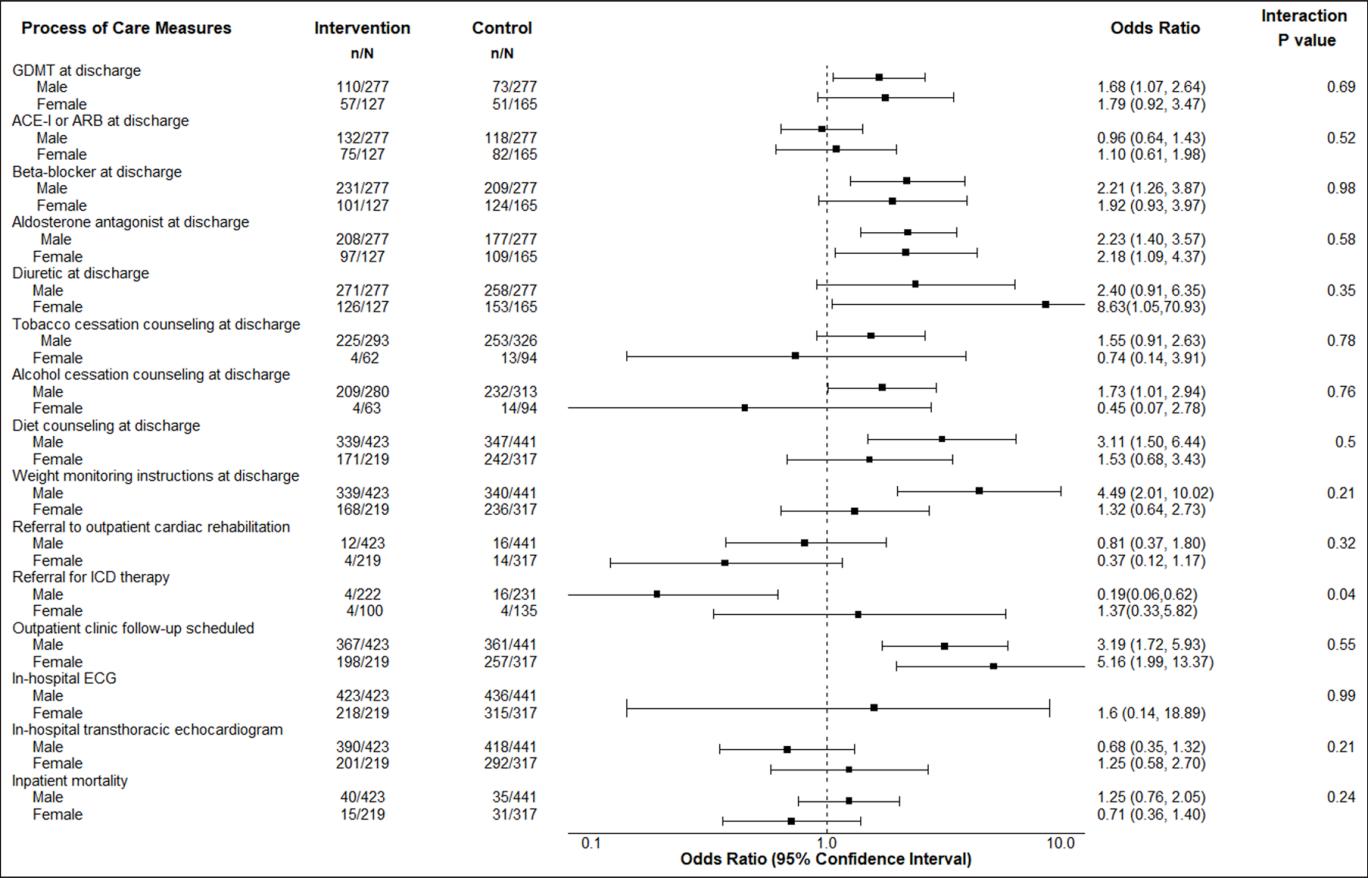
Sex-specific differences in the intervention effect for process of care measures and clinical outcomes. GDMT: guideline-directed medical therapy, ACE-I: angiotensin converting enzyme inhibitor, ARB: angiotensin receptor blocker, ICD: implantable cardioverter defibrillator, ECG: electrocardiogram.

Table 3 shows the crude differences in process of care measures and clinical outcomes stratified by sex between patients in the control and intervention periods. GDMT at discharge was observed in 110 (39.7%) male patients and 57 (44.9%) of female patients in the intervention period compared to 73 (26.4%) male patients and 51 (30.9%) female patients in the control group. The odds of GDMT at discharge were 79% higher among females (adjusted OR 1.79, 95% CI 0.92, 3.47) and 68% higher among males (adjusted OR 1.68, 95% CI 1.07, 2.64, p_interaction_ = 0.69, Figure 1, Supplementary Appendix Table 1). Female patients received lower rates of tobacco and alcohol cessation counseling at discharge compared to male patients (intervention period: 6.5% vs 76.8% tobacco cessation counseling, 6.3% vs 74.6% alcohol cessation counseling; p < 0.001 for both).

**Table 3:** Process of care measures and clinical outcomes of HF QUIK participants in control and intervention periods by sex.

	Control		P-value	Intervention		P-value
	
	Male n (%)	Female n (%)		Male n (%)	Female n (%)	

**Discharge process of care measures** ^1^						

GDMT at discharge^2^	73 (26.4)	51 (30.9)	0.29	110 (39.7)	57 (44.9)	0.33
ACE-I or ARB at discharge^2^	118 (42.6)	82 (49.7)	0.14	132 (47.7)	75 (59.1)	0.036
Beta-blocker at discharge^2^	209 (75.5)	124 (75.2)	0.98	231 (83.4)	101 (79.5)	0.35
Aldosterone antagonist at discharge^2^	177 (63.9)	109 (66.1)	0.62	208 (75.1)	97 (76.4)	0.78
Diuretic at discharge^2^	258 (93.1)	153 (92.7)	0.94	271 (97.8)	126 (99.2)	0.32
Tobacco cessation counseling^3^	253 (77.6)	13 (13.8)	<0.001	225 (76.8)	4 (6.5)	<0.001
Alcohol cessation counseling^3^	232 (74.1)	14 (14.9)	<0.001	209 (74.6)	4 (6.3)	<0.001
Diet counseling	347 (85.5)	242 (84.6)	0.13	339 (88.5)	171 (83.8)	0.11
Weight monitoring instructions	340 (83.7)	236 (82.5)	0.047	339 (88.5)	168 (82.4)	0.075
Referral to outpatient cardiac rehabilitation	16 (3.9)	14 (4.9)	0.54	12 (3.1)	4 (2.0)	0.40
Referral for ICD therapy^4^	16 (7.1)	4 (3.1)	0.12	4 (1.9)	4 (4.3)	0.22
Outpatient clinic follow-up scheduled	361 (88.9)	257 (89.9)	0.66	367 (95.8)	198 (97.1)	0.45
**In-hospital process of care measures**						

ECG	436 (98.9)	315 (99.4)	0.48	423 (100.0)	218 (99.5)	0.16
Transthoracic echocardiogram	418 (95.0)	292 (92.1)	0.10	390 (92.2)	201 (91.8)	0.85
**Clinical outcomes**						

Hospital length of stay, median (IQR), days	4.0 (3.0, 6.0)	4.0 (3.0, 7.0)	0.57	4.0 (3.0, 6.0)	4.0 (3.0, 6.0)	0.51
Inpatient mortality	35 (8.0)	31 (9.8)	0.37	40 (9.5)	15 (6.8)	0.26

^1^ Among participants discharged; N = 1279 with 692 in control period (406 male, 286 female) and 587 in intervention period (383 male, 204 female). ^2^ Among participants discharged with LVEF <40%; N = 846 with 442 in control period (277 male, 165 female) and 404 in intervention period (277 male, 127 female). ^3^ Among participants who responded to using tobacco or alcohol; for tobacco use (N = 775, control period (N = 420; 326 male, 94 female); intervention period (N = 355, 293 male, 62 female) and alcohol use (N = 750, control period (N = 407, 313 male; 94 female); intervention period (N = 343, 280 male; 63 female). ^4^ Among participants with LVEF ≤35%; N = 688 with 366 in control period (231 male, 135 female) and 322 in intervention period (222 male, 100 female). ACE-I: angiotensin converting enzyme inhibitor, ARB: angiotensin receptor blocker, GDMT: guideline-directed medical therapy, ICD: implantable cardioverter defibrillator, LVEF: left ventricular ejection fraction, ECG: electrocardiogram.

## Discussion

Among 1,400 patients admitted with HF in South India, we describe sex-specific differences in presentation, management, and in-hospital outcomes in a secondary analysis of a prospective interrupted time series study and explore sex-based differences in the effect of the quality improvement intervention. We observed that female HF patients were older, less likely to have comorbid chronic kidney disease, ischemic etiology of HF, and undergo coronary angiography and percutaneous coronary intervention during hospitalization compared to male patients. Importantly, there were no significant sex-specific differences in prescription of GDMT at hospital discharge using a HF-specific quality improvement toolkit, but overall rates remained suboptimal.

Female patients remain the minority of patients with HF enrolled in HF-specific studies in South Asia. Compared to 38% female patients in HF QUIK, The Trivandrum Heart Failure Registry enrolled 31% women in South India, the International Congestive Heart Failure (INTER-CHF) cohort study enrolled 38% women in India, and REPORT-HF enrolled 36% women from Southeast Asia [12,15,23,24]. Female patients with HFrEF are also under-represented in international, multi-center, randomized HF clinical trials [8]. This is partly attributed to HFrEF being more prevalent in males than females due to higher rates of macrovascular coronary artery disease and ischemic cardiomyopathy, but underrepresentation of females may also be related to underdiagnosis due to limited access to care [25,26]. Community-based observational cohorts demonstrate higher prevalence of HF with preserved ejection fraction (HFpEF) in females than males, postulated to be linked to higher rates of coronary microvascular dysfunction and HFpEF risk factors, including obesity in females [25,27,28]. Female HF patients in HF QUIK were older and had higher left ventricular ejection fraction than male HF patients, which is consistent with sex-specific differences in HF presentation globally [25,29]. Similarly, female patients with acute coronary syndromes were older as compared to male patients in large observational registries in India [9,10].

We observed no significant sex-specific differences in the effect of the quality improvement intervention on prescription of GDMT at hospital discharge between male and female HFrEF patients in the intervention period in HF QUIK. To our knowledge, no other in-hospital HF quality improvement intervention trials nor quasi-experimental studies have reported sex-specific outcomes [20]. Importantly, there were no significant sex-specific differences in prescription of GDMT at hospital discharge between male and female HFrEF patients in the control period in HF QUIK. In an analysis of 15,415 patients including 3,357 women from 55 countries enrolled in two recent large randomized clinical trials of pharmacological therapy in patients with HFrEF, there was no evidence of significant undertreatment of women with HFrEF with GDMT [8]. Although prescription of GDMT improved in both male and female patients in the intervention period compared to the control period, more than half of all eligible patients with HFrEF in HF QUIK remained sub-optimally treated. This is consistent with identified gaps in GDMT in ASIAN-HF registry with recommended target doses achieved in only 17% of those given ACEi or ARBs, 13% of those given beta-blockers, and 29% of those given aldosterone antagonists [30]. There were similarly low rates of GDMT in the GUIDE-IT randomized clinical trial with only 15% of patients achieving optimal therapy at six months with no sex-specific differences observed [31]. Prospective observational registry data suggests women have approximately 30% lower risk of death or heart failure hospitalization at 50% of the recommended doses of ACEi or ARBs and beta-blockers, with no further benefit at higher dose levels [32]. Pragmatic implementation strategies, such as a HFrEF polypill using fixed-dose combinations of GDMT with titration algorithms, have been proposed to bridge the gap between clinical guidelines and clinical practice in undertreated populations [33]. Clinical trials evaluating efficacy and safety of HFrEF polypills will need to pay careful attention to sex-specific differences in optimal dosing of GDMT and clinical outcomes [32,34].

Multilevel implementation strategies targeting various sex-specific barriers at the patient, provider, and health-system level will be needed to narrow the GDMT treatment gap in India. Previous research in Kerala illustrates many community physicians discontinue GDMT during outpatient follow-up after hospitalization for HF due to concerns of side effects including relative hypotension [21]. A secondary analysis of The Guiding Evidence-Based Therapy Using Biomarker Intensified Treatment (GUIDE-IT) trial demonstrated therapeutic inertia to be a key barrier in achieving optimal GDMT rates [31]. Qualitative research exploring self-care behaviors of patients with HF via in-depth interviews (n = 22, 41% female) in South India suggests most female HF patients were passive recipients of health information and frequently relied on a male caregiver (husbands or sons) to oversee HF management including fluid/diet restrictions and medication administration [35]. Further study is needed to understand whether sex-specific HF behavioral interventions may complement existing and new implementation strategies to improve the GDMT treatment gap.

This study has several strengths. This is the first quasi-experimental study evaluating the effect of an in-hospital quality improvement intervention for patients with HF in a low- or middle-income country to assess sex-specific presentation, management, and outcomes [20]. We enrolled 536 (38.3%) female HF patients, which is higher than other representative HF studies in the region [15,24]. Significant formative research including a systematic review and key informant in-depth interviews including eight (38%) female participants were done to inform the design of the quality improvement intervention [20,21]. This study has several key limitations including the *post hoc* nature of the analyses, moderate sample size and power to detect potential differences, and a study design that did not prospectively seek to detect sex-specific differences. Furthermore, we are unable to comment on adherence to GDMT as patients were not followed after hospital discharge. However, this study highlights sex-based differences among patients hospitalized with HF in India, a region with limited data on sex-based differences in cardiovascular disease.

## Conclusion

This secondary analysis of a quasi-experimental study in Kerala demonstrates female HF patients were older, less likely to have comorbid chronic kidney disease, ischemic etiology of HF, and undergo coronary angiography and percutaneous coronary intervention during hospitalization compared to male patients with HF. There were no significant sex-specific differences in the effect of the quality improvement intervention on prescription of GDMT in patients with HFrEF at hospital discharge. Both male and female patients with HFrEF remained undertreated in the intervention period, demonstrating the need for implementation strategies to close the HFrEF treatment gap in South India.

## Additional Files

The additional files for this article can be found as follows:

10.5334/gh.1043.s1 Supplementary Appendix Figure 1. Flowchart of HF QUIK patients.

10.5334/gh.1043.s2 Supplementary Appendix Table 1. Unadjusted and adjusted odds of process of care measures and clinical outcomes among HF QUIK participants by sex.
