# Supplementary material for: Presentation, Management, and In-Hospital Outcomes of Patients with Acute Heart Failure in South India by Sex: A Secondary Analysis of a Prospective, Interrupted Time Series Study
Source: Glob Heart. 2021 Sep 27;16(1):63. doi: 10.5334/gh.1043 (PMC8485866; doi:10.5334/gh.1043)

Presentation, Management, and In-Hospital Outcomes of Patients with Acute Heart Failure in South India by Sex: A Secondary Analysis of a Prospective, Interrupted Time Series Study

Supplementary Appendix

Figure 1. Flowchart of HF QUIK patients.

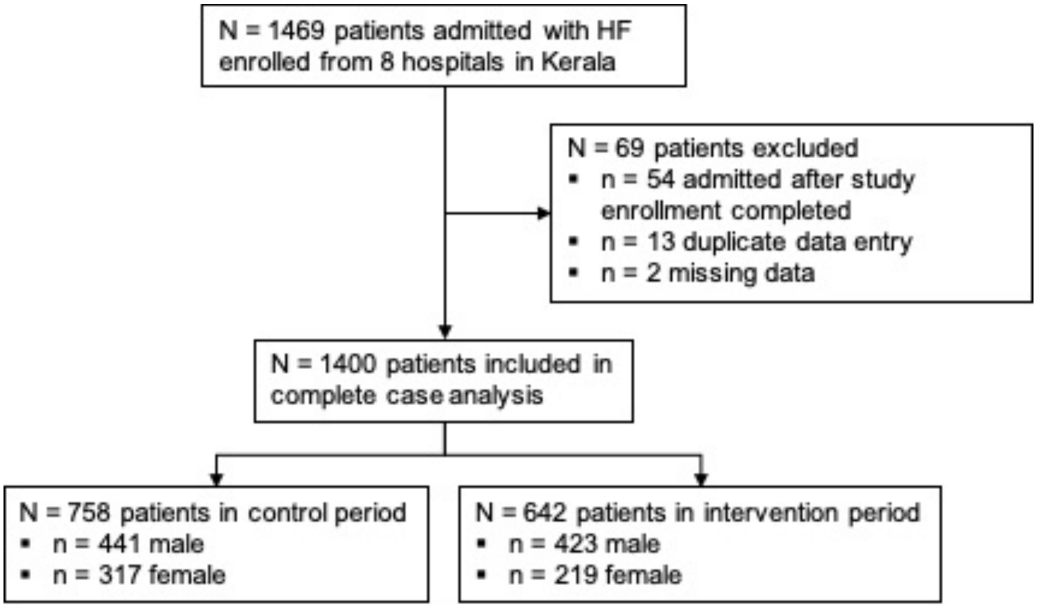

Supplement: Supplementary Appendix Figure 1. — Flowchart of HF QUIK patients. [file gh-16-1-1043-s1.pdf]
