# Supplementary material for: Presentation, Management, and In-Hospital Outcomes of Patients with Acute Heart Failure in South India by Sex: A Secondary Analysis of a Prospective, Interrupted Time Series Study
Source: Glob Heart. 2021 Sep 27;16(1):63. doi: 10.5334/gh.1043 (PMC8485866; doi:10.5334/gh.1043)
Supplement: Supplementary Appendix Table 1. — Unadjusted and adjusted odds of process of care measures and clinical outcomes among HF QUIK participants by sex. [file gh-16-1-1043-s2.pdf]

Table 1. Unadjusted and adjusted odds of process of care measures and clinical outcomes among HF QUIK participants by sex.

	OR (95% CI)				Interaction P value
	Female		Male		
	Unadjusted	Adjusted[1]	Unadjusted	Adjusted[1]	
**Discharge process of care measures**[2]					
GDMT at discharge[3]	1.80 (1.12,2.92)	1.79 (0.92,3.47)	1.83 (1.28,2.62)	1.68 (1.07,2.64)	0.698
ACE-I or ARB at discharge[3]	1.44 (0.90,2.30)	1.10 (0.61,1.98)	1.23 (0.88,1.72)	0.96 (0.64,1.43)	0.523
Beta-blocker at discharge[3]	1.25 (0.72,2.19)	1.92 (0.93,3.97)	1.61 (1.06,2.45)	2.21 (1.26,3.87)	0.977
Aldosterone antagonist at discharge[3]	1.63 (0.97,2.75)	2.18 (1.09,4.37)	1.69 (1.17,2.43)	2.23 (1.4,3.57)	0.582
Diuretic at discharge[3]	9.06 (1.15,71.12)	8.63 (1.05,70.93)	3.15 (1.23,8.06)	2.40 (0.91,6.35)	0.351
Tobacco cessation counseling[4]	0.43 (0.13,1.38)	0.74 (0.14,3.91)	0.95 (0.66,1.39)	1.55 (0.91,2.63)	0.784
Alcohol cessation counseling[4]	0.39 (0.12,1.24)	0.45 (0.07,2.78)	1.03 (0.71,1.49)	1.73 (1.01,2.94)	0.763
Diet counseling	0.92 (0.56,1.51)	1.53 (0.68,3.43)	1.27 (0.83,1.93)	3.11 (1.50,6.44)	0.505
Weight monitoring instructions	0.97 (0.6,1.56)	1.32 (0.64,2.73)	1.45 (0.96,2.19)	4.49 (2.01,10.02)	0.216
Referral to outpatient cardiac rehabilitation	0.39 (0.13,1.19)	0.37 (0.12,1.17)	0.79 (0.37,1.69)	0.81 (0.37,1.80)	0.320
Referral for ICD therapy[5]	1.41 (0.34, 5.79)	1.37 (0.33, 5.82)	0.25 (0.08, 0.76)	0.19 (0.06, 0.62)	0.037
Outpatient clinic follow-up scheduled	3.60 (1.46,8.85)	5.16 (1.99,13.37)	2.80 (1.55,5.05)	3.19 (1.72,5.93)	0.555
**In-hospital process of care measures**					
ECG	1.38 (0.12,15.36)	1.60 (0.14,18.89)	na	na	0.988
Transthoracic echocardiogram	0.96 (0.51,1.80)	1.25 (0.58,2.70)	0.62 (0.36,1.09)	0.68 (0.35,1.32)	0.213
**Clinical outcomes**					
Inpatient mortality	0.68 (0.36,1.28)	0.71 (0.36,1.40)	1.21 (0.75,1.94)	1.25 (0.76,2.05)	0.244

[1]Adjusted for age and random cluster effect for hospital.
[2]Among participants discharged; N = 1279 with 692 in control period (406 male, 286 female) and 587 in intervention period (383 male, 204 female)
[3]Among participants discharged with LVEF <40%; N = 846 with 442 in control period (277 male, 165 female) and 404 in intervention period (277 male, 127 female)
[4]Among participants who reported tobacco or alcohol use; for tobacco use (N=775, control period (N=420; 326 male, 94 female); intervention period (N=355, 293 male, 62 female) and alcohol use (N=750, control period (N=407, 313 male, 94 female); intervention period (N=343, 280 male; 63 female)
[5]Among participants with LVEF ≤35%; N = 688 with 366 in control period (231 male, 135 female) and 322 in intervention period (222 male, 100 female)

GDMT: guideline-directed medical therapy, ACE-I: angiotensin converting enzyme inhibitor, ARB: angiotensin receptor blocker, ICD: implantable cardioverter defibrillator, LVEF: left ventricular ejection fraction, ECG: electrocardiogram
